# Spatial patterns of reproduction suggest marginal habitat limits continued range expansion of black bears at a forest‐desert ecotone

**DOI:** 10.1002/ece3.10658

**Published:** 2023-10-31

**Authors:** Sean M. Sultaire, Robert A. Montgomery, Patrick J. Jackson, Joshua J. Millspaugh

**Affiliations:** ^1^ Wildlife Biology Program University of Montana Missoula Montana USA; ^2^ Department of Biology University of Oxford Oxford UK; ^3^ Nevada Department of Wildlife Reno Nevada USA

**Keywords:** black bear reproduction, center–periphery hypothesis, Great Basin, multistate occupancy model, piñon‐juniper woodland, range boundary, *Ursus americanus*

## Abstract

Investigating spatial patterns of animal occupancy and reproduction in peripheral populations can provide insight into factors that form species range boundaries. Following historical extirpation, American black bears (*Ursus americanus*) recolonized the western Great Basin in Nevada from the Sierra Nevada during the late 1900s. This range expansion, however, has not continued further into the Great Basin despite the presence of additional habitat. We aimed to quantify whether reduced reproduction toward the range edge contributes to this range boundary. We analyzed black bear detections from 100 camera traps deployed across black bear distribution in western Nevada using a multistate occupancy model that quantified the probability of occupancy and reproduction (i.e., female bears with cubs occupancy) in relation to changes in habitat type and habitat amount toward the range boundary. We detected a strong effect of habitat amount and habitat type on the probability of black bear occupancy and reproduction. At similar levels of landscape‐scale habitat amount (e.g., 50%), estimated probability of occupancy for adult bears in piñon‐juniper woodlands near the range boundary was 0.39, compared to ~1.0 in Sierra Nevada mixed‐conifer forest (i.e., core habitat). Furthermore, estimated probability of cub occupancy, conditional on adult bear occupancy, in landscapes with 50% habitat was 0.32 in Great Basin piñon‐juniper woodlands, compared to 0.92 in Sierra Nevada mixed‐conifer forest. Black bear range in the western Great Basin conforms to the center–periphery hypothesis, with piñon‐juniper woodland at the range edge supporting ecologically marginal habitat for the species compared to habitat in the Sierra Nevada. Further geographic expansion of black bears in the Great Basin may be limited by lower occupancy of reproducing females in piñon‐juniper woodland. Center–periphery range dynamics may be common in large carnivore species, as their dispersal ability allows them to colonize low‐quality habitat near range edges.

## Graphical abstract

Investigating spatial patterns of reproduction at range boundaries provides insight into factors limiting species distributions. We quantified American black bear reproduction across differences in habitat quality near a regional range boundary, finding strong evidence for lower habitat quality in the periphery of their range in the Great Basin of western North America.

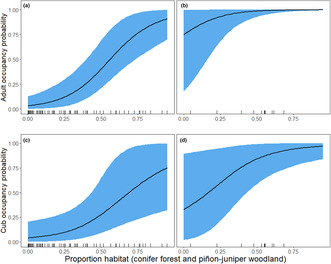

## 1 INTRODUCTION

Species range boundaries are attributable to a diversity of processes including unsuitable climate conditions, interspecific interactions, and habitat availability (Gaston, 2009; Sexton et al., 2009). The center–periphery hypothesis, for example, predicts that peripheral portions of species ranges are ecologically marginal when compared to habitat closer to the core (Brown, 1984). Consequently, occupancy, abundance, population performance (i.e., fitness), and genetic diversity are all expected to be lower for populations at the edge of a species range (Brown, 1984; Pironon et al., 2017). Although lower genetic diversity in peripheral populations has been detected with considerable evidence (Eckert et al., 2008; Malaney et al., 2018; Pironon et al., 2017), support for the prediction of lower abundance at range peripheries has been mixed (Dallas et al., 2017; Fristoe et al., 2022; Santini et al., 2019). There remains little evidence for declines in the demographic performance of populations toward range boundaries (Pironon et al., 2017). This evidence indicates that habitats at the edge of species ranges are not always of comparatively lower quality, highlighting the importance of relating population performance measures to environmental gradients near range boundaries when investigating the center–periphery hypothesis (Eckhart et al., 2011). For example, topographic complexity and associated environmental gradients can create large changes in environmental suitability for species over small geographic areas of their range that obscures center–periphery patterns across species ranges (Fristoe et al., 2022). However, species range boundaries that occur in topographically complex regions also provide opportunities to investigate whether the range of a species adheres to the center–periphery hypothesis across relatively small geographic areas.

Due to source–sink dynamics, the presence of reproductive individuals often better reflects the quality of habitat at a location compared to species occupancy or abundance (Pulliam, 1988; Van Horne, 1983). Among species of large mammals, parental investment and coupled energetic expenditures tend to be high, and reproduction is often not undertaken under conditions of resource scarcity (Harlow et al., 2002; Samson & Huot, 1995). As a result, investigating patterns of large mammal reproduction in relation to environmental gradients near range boundaries may reveal whether lower habitat quality in peripheral areas limits their distribution. Evidence for lower occupancy of reproductive individuals near range boundaries would support declines in demographic performance, as predicted under the center–periphery hypothesis (Pironon et al., 2017). As peripheral populations may be most sensitive to global change (i.e., climate change; Sexton et al., 2009), in addition to improving our theoretical understanding of range boundary formation, quantifying factors limiting large mammal populations at range edges can inform species conservation efforts.

American black bears (*Ursus americanus*) are a large mammalian carnivore with an extensive range spanning most of temperate and boreal North America (Larivière, 2001). They are primarily a forest‐dwelling species, with their range naturally fragmented by the availability of forest cover across the western United States and more recently fragmented in the eastern United States by agriculture and development (Laliberte & Ripple, 2004). Black bears have a comparatively slow life history with females taking 2 years to raise cubs to maturity, and reproductive attempts closely tied to resource availability (Beckmann & Berger, 2003a; Costello et al., 2003). Black bears are also highly mobile, with individual home ranges of several hundred km^2^ documented (Ditmer et al., 2018) and occasional movement distances of >280 km in a year (Liley & Walker, 2015). Females with cubs, however, tend to have smaller home ranges (Ditmer et al., 2018; Gantchoff et al., 2019), although this can vary seasonally (Moyer et al., 2007). The smaller home ranges of female black bears with cubs and the importance of resource availability to reproductive attempts suggest that cub occupancy is more indicative of the habitat quality at a location than the presence of individual adult bears that may be transient. Hence, quantifying spatial patterns of black bear reproduction near range boundaries can elucidate how reductions in habitat quality limit the species distribution. As black bears are one of the few species of large carnivores that have increased in abundance and distribution in the 21st century (Ripple et al., 2014), information on spatial patterns of reproduction in recently expanded populations can help assess the future trajectory of these populations.

**FIGURE 1:**
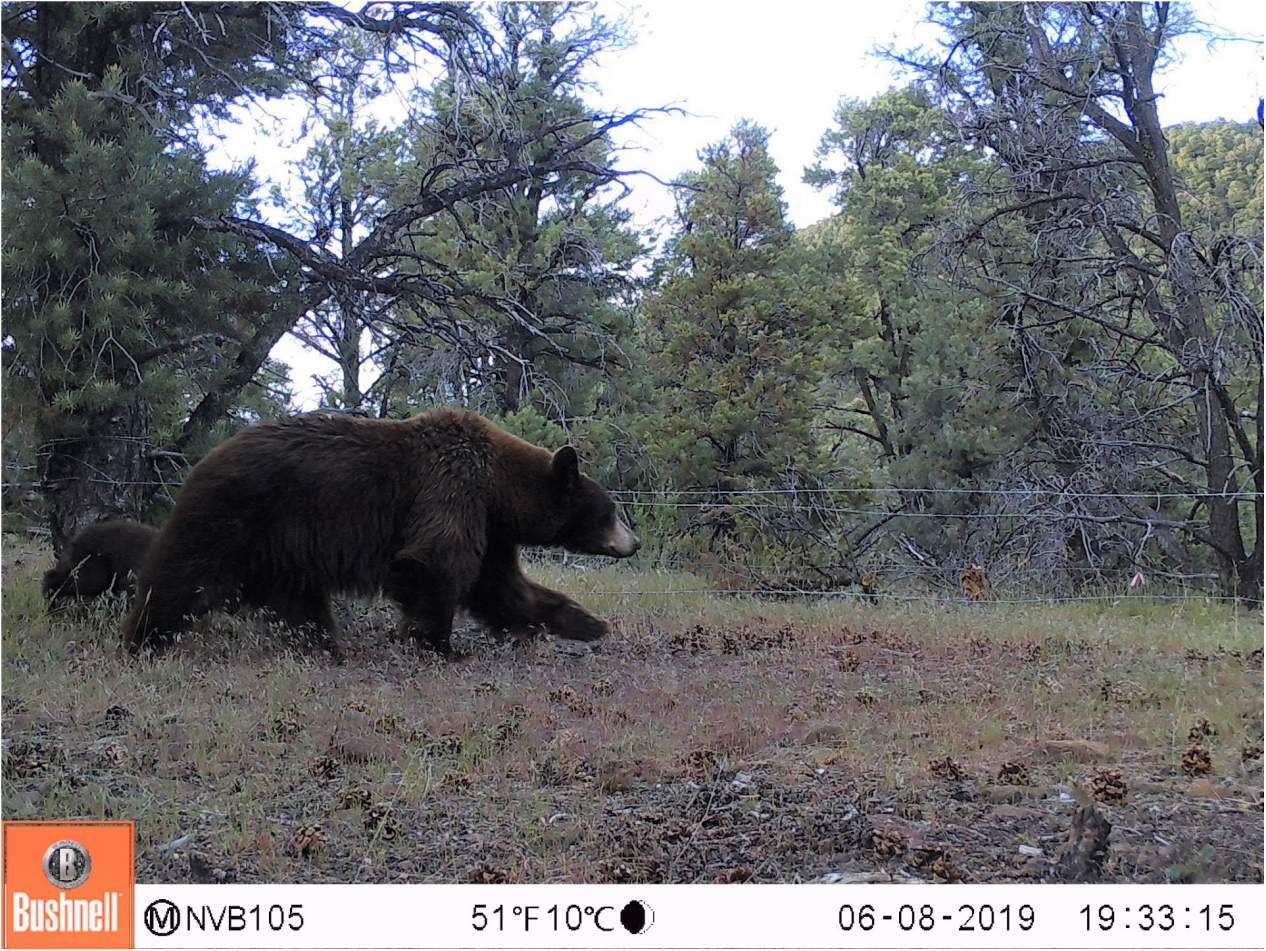
A camera‐trap photo of a female black bear with a single cub (lower left) visiting a sampling location within piñon‐juniper woodland in the Great Basin of Nevada. In this manuscript, we provide evidence that these woodlands are marginal habitat for black bears, supporting lower probability of occupancy for females with cubs compared to conifer forests in the nearby Sierra Nevada.

**FIGURE 2:**
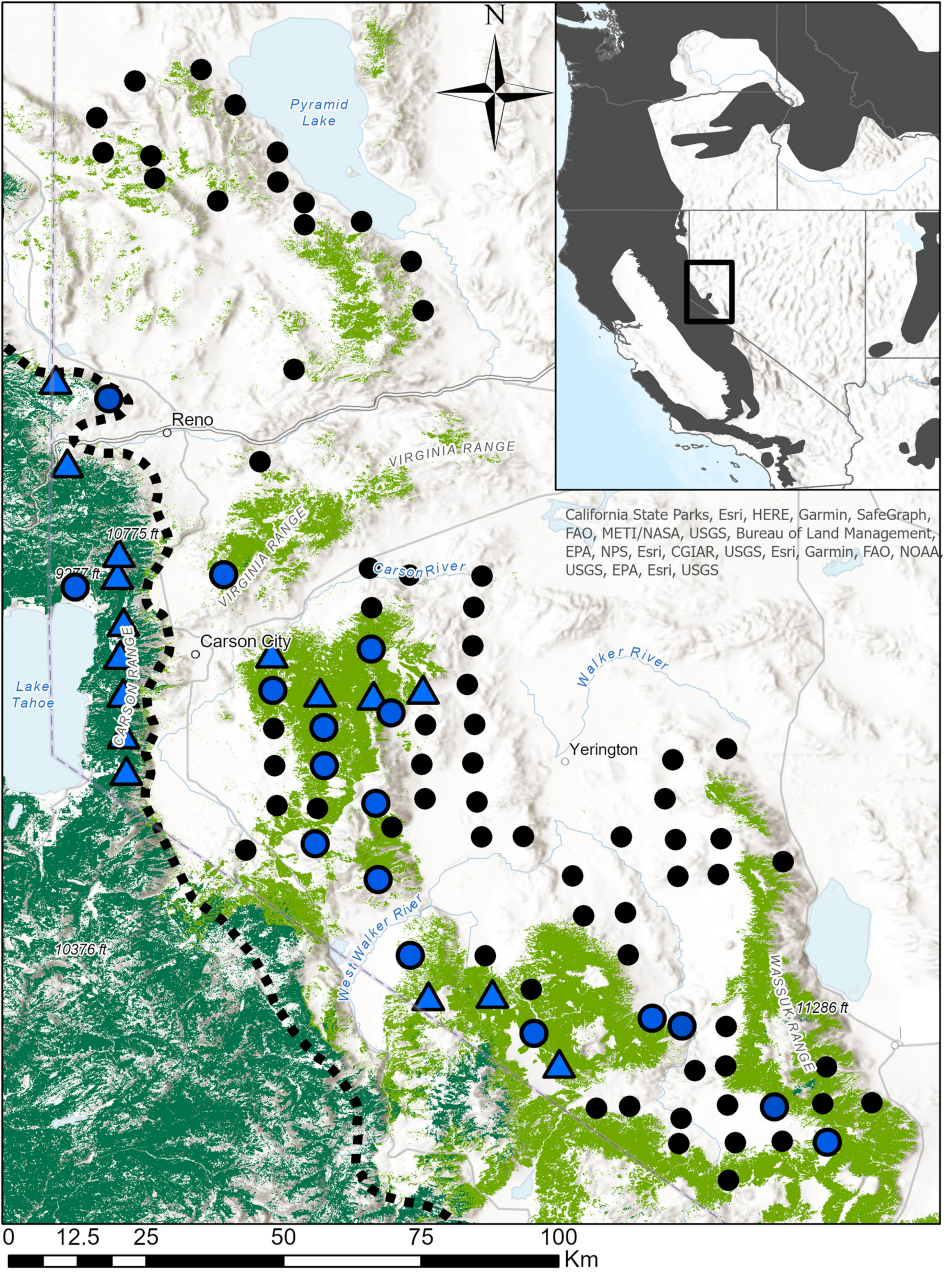
Study area map showing the location of 100 study sites where motion‐activated cameras were deployed (points) at the ecotone between the Sierra Nevada and the Great Basin Desert (dashed line). Blue points indicate the 33 sites where we detected black bears on cameras and the triangles further indicate the 16 of those sites where cubs were detected. The distribution of piñon‐juniper woodlands is shown in light green and that of the mixed‐conifer forest in dark green. The inset map shows the location of our study area in the context of American black bear range in western North America and that our study area straddles the eastern boundary of the Sierra Nevada black bear population. Credit USDA/DOI LANDFIRE database for land cover data (https://www.landfire.gov/evt.php).

Multistate site occupancy models are useful analytical tools for investigating spatial patterns of species occupancy and reproduction (Gould, Ray, et al., 2019; MacKenzie et al., 2009; Martin et al., 2009). These models separate the observation and detection process, providing estimates of species occupancy and reproduction conditional on occupancy that are corrected for imperfect detection, as well as the environmental factors that influence these parameters (Nichols et al., 2007). Using a multistate occupancy model, we analyzed black bear detections from a network of motion‐activated cameras (i.e., camera traps) deployed across a recolonized area of the species range in the western Great Basin. Female bears with cubs are easily identified in camera‐trap photos (Figure 1; Fisher et al., 2014), allowing us to use this method to quantify spatial patterns black bear reproduction at this range boundary. The range boundary coincides with an ecotone between mixed‐conifer forest in the Sierra Nevada, and cold desert in the Great Basin (Figure 2; Charlet, 1996) where black bears occupy mountain ranges with piñon‐juniper woodland in this transition zone. Mixed‐conifer forest in the Sierra Nevada represents more typical black bear habitat in their western range, particularly California, with mast‐producing bear foods including manzanita (*Arctostaphylos* sp.), huckleberry oak (*Quercus vaccinifolia*), and whitebark pine (*Pinus albicaulis*). These food sources are largely absent in the Great Basin, but piñon pine (*Pinus monophylla*) produces nutritious nuts that are abundant during years of heavy masting (Redmond et al., 2012). Black bear density and habitat use are positively correlated to measures of primary productivity in their western range (Gould, Gould, et al., 2019; Welfelt et al., 2019), so there is reason to expect that occupancy of females with cubs likely also declines across gradients in productivity such as those found in our study area.

Due to the wide‐ranging nature of adult black bears (particularly males), we predicted a stronger trend toward lower occupancy of female black bears with cubs than adult bears without cubs at the range edge. We further predicted that reductions in occupancy of female black bears with cubs near the range boundary would be related to both reductions in habitat amount and habitat quality, the latter in the form of a shift from mixed‐conifer forest to piñon‐juniper woodland. These predictions are consistent with the predictions of the center–periphery hypothesis that habitat on the edge of black bear range in the Great Basin is ecologically marginal for the species and supports lower levels of reproduction than core habitats further from the range boundary.

## 2 METHODS

### 2.1 Study area

We conducted this study in western Nevada, at the ecotone between the Sierra Nevada and the Great Basin Desert (Figure 2). Rain shadowing by the Sierra Nevada results in sharp decreases in precipitation from west to east across the study area, with equally sharp transition from mixed‐conifer forest (primarily *Pinus* and *Abies* sp.) to shrub‐steppe vegetation (*Artemisia*, *Chrysothamnus* sp.) along this precipitation gradient. Mountain ranges within this transition with intermediate precipitation support piñon‐juniper woodland, primarily comprised of *Pinus monophylla* and *Juniperus osteosperma*. Average annual precipitation varies from 1400 mm/year in the higher elevations of the Sierra Nevada to 180 mm in low elevation desert (PRISM Climate Group, 2021). The region is mountainous with elevations ranging from approximately 1100 to 3400 m. Black bears recolonized the semi‐arid mountain ranges in the Great Basin from refugial populations in the Sierra Nevada during the late 1900s (Lackey et al., 2013; Malaney et al., 2018); however, their range has been fairly static since the early 2000s (Sultaire et al., 2023). The greater Sierra Nevada population could be considered a peripheral extension of black bear range from the north where populations are more continuous. In this study, however, we conceptualized the Sierra Nevada as part of the core bear range, and the Great Basin as peripheral bear range. Reno, Nevada, is a large urban area located in the northern portion of the study area (human population ~280,000 in 2020).

### 2.2 Site selection and field methods

We selected 100 sites to deploy camera traps using a 49‐km^2^ grid‐based sampling (7 × 7 km grid cells) scheme that spanned the distribution of black bears in Nevada as defined by the Nevada Department of Wildlife. Gaps within the grid resulted from private lands and federally designated Wilderness areas, where permission to deploy cameras could not be granted (Figure 2). Starting in May 2018, we deployed camera traps (Bushnell Trophy Cam HD, Model 119776C) at the center of each 49‐km^2^ grid cell approximately 1 m above ground level. If the grid cell center was inaccessible because of prevailing topographical changes in this rugged landscape, we placed the camera at the closest, accessible location. In forest and woodland locations, we secured cameras to tree trunks, and in treeless areas, we installed metal fence posts to mount cameras. To increase the time cameras could operate without being checked, we set cameras to take three photos each time they were triggered and subsequently deactivate for 10 min (Lepard et al., 2018). During the summer months (June–August 2018–19, July–September 2020) sites were visited every 7–10 days to retrieve photos from cameras and apply a scent attractant within the viewshed of the camera. During this time, barbed‐wire hair snags were also deployed in camera viewsheds to collect hair for genetic identification of individuals (Sultaire et al., 2023). Because we could not distinguish adults and cubs from genetic data, we do not consider this data source in this manuscript. Cameras were operational but not checked during the remaining 9 months of the year, which included hibernation when bears were not detectable. Across 3 years of sampling, we observed only one female black bear moving among sampling locations with genetic data, indicating that sites were reasonably independent of each other with respect to female space use.

### 2.3 Multistate occupancy model

We manually identified all photos containing black bears, separately classifying all photos with cubs as an indication of reproduction occurring at a site. We considered black bear reproduction to occur at a site if we observed either first‐ or second‐year cubs in a photo, regardless of whether the female bear was also captured in the photo. Although females are usually in close proximity to cubs, due to the 10‐min delay period, there were instances where only cubs were detected. Body size is generally a reliable way to distinguish second‐year cubs from older bears (Marks & Erickson, 1966) but if there was any ambiguity as to whether the bear in a photograph was a cub, we classified the detection as an adult bear. Multistate occupancy models can handle misidentification of the highest occupancy state (cub occupancy) as the lower occupied state (adult occupancy) but not vice versa (Nichols et al., 2007). Instances of cameras detecting a female bear but not her cubs likely lowered the cub detection probability estimate (see below) but did not violate model assumptions. About 50% of cub photos did not capture the adult female bear, with about half of these detections considered first‐year cubs and half second‐year cubs. We did not differentiate between first‐ and second‐year cubs in the analysis. We organized adult bear and cub detections into monthly detection histories for the nine‐month period bears are typically active each year (Mar–Nov), resulting in nine sampling periods per year. Hence, estimates of occupancy and reproduction probability corresponded to the probability that black bear adults or cubs use a camera‐trap location at least once during 9‐month primary sampling seasons (Lele et al., 2013). Detection probability quantified the probability that black bear adults or cubs use a camera site at a 30‐day temporal resolution.

We treated detection histories from each site for each year as separate observations and analyzed the data in a Bayesian multistate occupancy model with site‐specific random effects on occupancy and reproduction parameters to account for repeat measures from the same sites. We further included two fixed‐effect covariates on occupancy and reproduction. One covariate was continuous and measured the proportion of land cover comprised of black bear habitat (mixed‐conifer forest or piñon‐juniper woodland) within a 5‐km radius of sites, derived from the USDA/USDOI LANDFIRE database (https://www.landfire.gov/evt.php). The second covariate was a categorical covariate for ecoregion, which indicated whether a site was located in the Sierra Nevada ecoregion (*n* = 11) or the Great Basin ecoregion (*n* = 89; Figure 2), derived from U.S. EPA ecoregion dataset (Omernik & Griffith, 2014). As bears are closely tied to conifer forest and woodland in the study area, the habitat covariate captured the effect of habitat availability on the probability of black bear occupancy and reproduction. Due to the sharp transition from mixed‐conifer forest in the Sierra Nevada to less productive piñon‐juniper woodland in the Great Basin, the ecoregion covariate captured changes in black bear habitat quality on probability of occupancy and reproduction. Although proportion habitat was on average higher in the Sierra Nevada, sites in the Great Basin spanned the full gradient of habitat amount in the surrounding landscape (see Section 3), and we were able to include both covariates in the same model. Hence, the occupancy and reproduction levels of multistate occupancy model took the same form:

$$\psi_{i}=\alpha_{\text{1ψ}}+\alpha_{2i\psi }+{\beta_{\text{1ψ}}}^{*}\;\text{habitat}5{\mathrm{km}}_{i}+{\beta_{\text{2ψ}}}^{*}\;{\text{Ecoregion}}_{i};$$

$$R_{i}=\alpha_{\text{1R}}+\alpha_{2iR}+{\beta_{\text{1R}}}^{*}\;\text{habitat}5{\mathrm{km}}_{i}+{\beta_{\text{2R}}}^{*}\;{\text{Ecoregion}}_{i}$$

with *ψ*_*i*_ representing the probability site_*i*_ is occupied by adult black bears; *R*_*i*_ representing the probability site_*i*_ is occupied by cubs, conditional on adult occupancy. *α*_1ψ_ and *α*_1R_ are global intercept terms and *α*_*2i*ψ_ and *α*_*2i*R_ were site‐specific random effects on occupancy and reproduction, respectively. *β*_1ψ_ and *β*_1R_ were the coefficients for the effect of proportion habitat (forest and woodland) on adult and cub occupancy, respectively, and *β*_2ψ_ and *β*_2R_ were the coefficients for the effect of ecoregion on adult and cub occupancy. The Great Basin ecoregion was specified as the intercept for the ecoregion covariates, and therefore quantified differences in adult and cub occupancy probability in the Sierra Nevada compared to the Great Basin.

We used a multinomial formulation of multistate occupancy models (Nichols et al., 2007), which estimated three different detection probabilities: (i) the probability of detecting an adult bear conditional on their occupancy, (ii) the probability of detecting an adult conditional on cubs occupying a site, and (iii) the probability of detecting cubs conditional on whether they occupy a site. Occupancy models assume that false positive detections do not occur and each of these conditional probabilities are zero if the state they estimate detection for does not occur at a site. In addition to effects of habitat at coarse extent on occupancy (i.e., probability of site use at least once; Lele et al., 2013), we predicted that sites with more habitat at smaller spatial scales would have more frequent use by black bears. To capture potential heterogeneity in detection probability driven by variation in black bear site use frequency, as measured at the temporal resolution of 30‐day secondary periods, we included the effect of proportion habitat (conifer forest and piñon‐juniper) within a 1‐km buffer of sites on detection probability (Sultaire et al., 2023). The three detection submodels all took the form:

$$p_{\mathrm{it}}=\alpha +{\beta_{1}}^{*}\;\text{habitat}1{\mathrm{km}}_{i}$$

where *p*_it_ represents the three separate detection probabilities for each occupancy state at site *i* and survey period *t*, *α* was the intercept term, *β*_1_ was the coefficient for effect of local habitat proportion on detection probability of each occupancy state, and habitat1km_*i*_ was the proportion habitat (mixed‐conifer forest or piñon‐juniper woodland) within 1 km of site *i*. The continuous occupancy and detection covariates were standardized to a mean of zero and standard deviation of one for model fitting. We used uninformative priors for each variable in the model, which we adapted from multistate occupancy code provided in Kéry and Royle (2020). The Bayesian model was implemented using JAGS software via the R package jagsUI *version* 1.5.2 (Kellner, 2021). We ran three MCMC chains for 200,000 iterations, a thinning rate of 40, and discarded the first 100,000 runs as burn‐in. These MCMC settings resulted in 2500 iterations per chain and 7500 total iterations contributing to the posterior distribution of each parameter. We assessed convergence between the three chains using the Gelman‐Rubin statistic (i.e., *Rhat*) and considered parameters with Rhat values <1.1 to have converged across the three chains. Code used to fit Bayesian multistate occupancy model is also available in Appendix S1.

## 3 RESULTS

Over the 30‐month duration of the study, our grid totaled 63,636 camera‐trap nights. During these camera‐trap nights, we detected black bears 510 times at 33 sites including all 11 sites in the Sierra Nevada (Figure 1). Black bear cubs were detected 44 times at 16 sites, 10 of which were in the Sierra Nevada. The detection probability of adult black bears increased with the proportion habitat within 1 km of survey sites (Table 1), varying from 0.11 to 0.21 across the observed gradient. In contrast, the detection of cubs and the detection of adults when cubs occupied a site did not increase with proportion habitat within 1 km of sites (Table 1). Estimated monthly detection probability of cubs was 0.23 (95% CI = 0.11, 0.42) and estimated detection probability for adult bears when cubs occupied a site was 0.48 (95% CI = 0.32, 0.63).

**TABLE 1:** Coefficient estimates on the logit scale and 95% credible intervals for the effects of covariates on black bear adult occupancy, reproduction (i.e., cub occupancy), and detection from a Bayesian multistate occupancy model.

	Occupancy	Reproduction	p22^a^	p23^b^	p33^c^
Ecoregion	**5.8 (3.0, 9.3)**	**3.3 (1.0, 6.2)**	–	–	–
Habitat	**2.1 (1.0, 3.5)**	**1.7 (0.3, 2.8)**	**0.8 (0.1, 1.6)**	0.0 (−0.5, 0.5)	−0.1 (−0.8, 0.6)

*Note*: Proportion habitat was summarized within a 5‐km radius for occupancy and reproduction covariates and a 1‐km radius of sites for detection covariates. Conifer covariates were standardized with a mean of zero and standard deviation of one. Bold values indicate effects for which the 95% credible interval did not include zero. The occupancy and reproduction parameters also included site‐level random intercepts, with estimated precision of 0.45 for adult occupancy and 1.97 for reproduction (posterior medians).

^a^ Detection probability of adult bears in the absence of cubs.

^b^ Detection probability of adult bears if cubs are present.

^c^ Detection of cubs if they are present.

**FIGURE 3:**
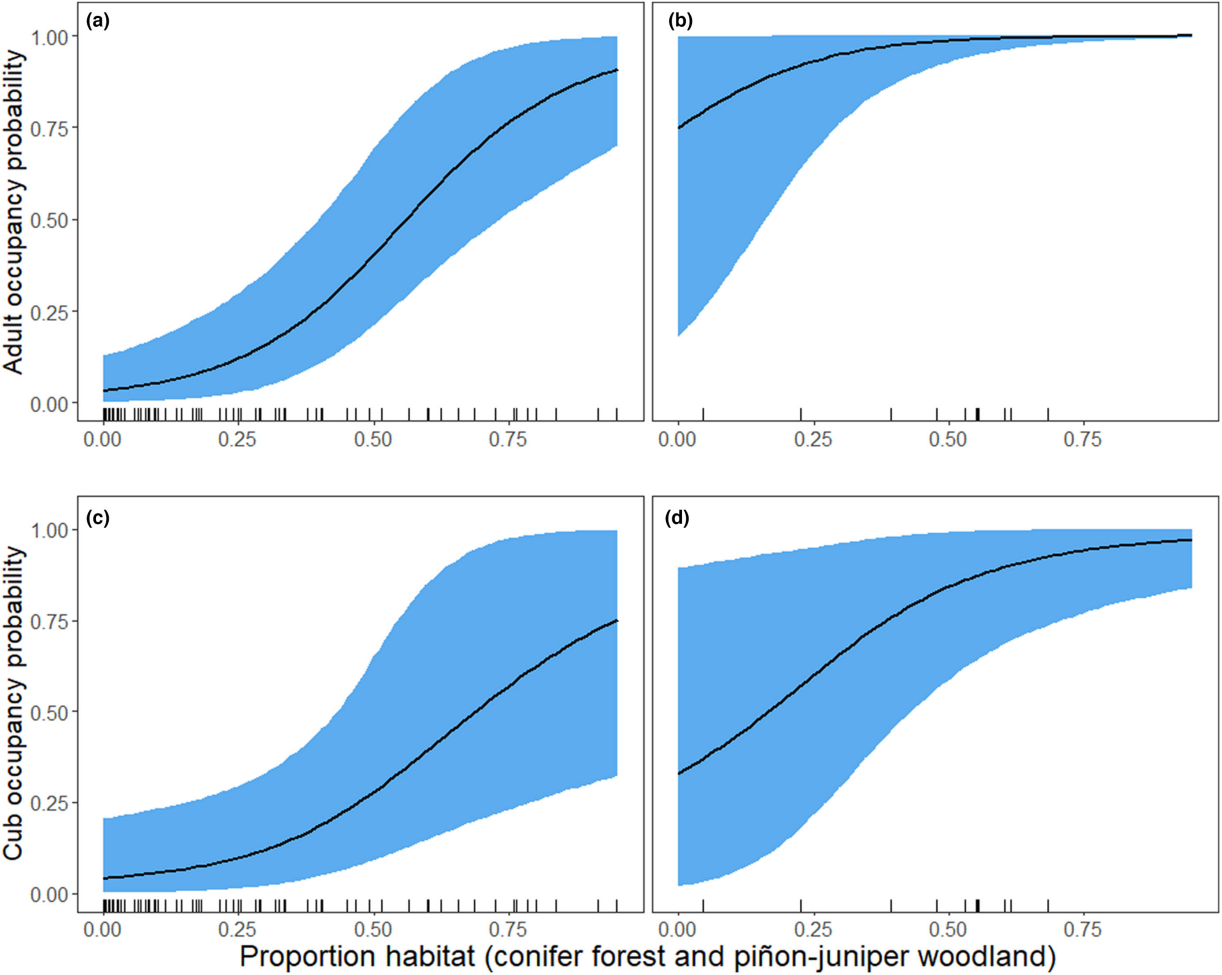
Predicted relationships between proportion habitat (conifer forest and woodland) and (a) adult black bear occupancy probability in the Great Basin ecoregion, (b) adult black bear occupancy probability in the Sierra Nevada, (c) cub occupancy probability (conditional on adult occupancy) in the Great Basin, and (d) cub occupancy probability (conditional on adult occupancy) in the Sierra Nevada. The black lines represent the mean predicted relationships and the blue shaded areas represent the 95% credible intervals for the predictions. Ticks on *X*‐axis represent proportion habitat (conifer forest and woodland) at each site in each ecoregion. Predictions generated from a multistate occupancy model fit to 3 years of black bear detection data on camera traps, collected from 100 sites, 2018–2020.

**FIGURE 4:**
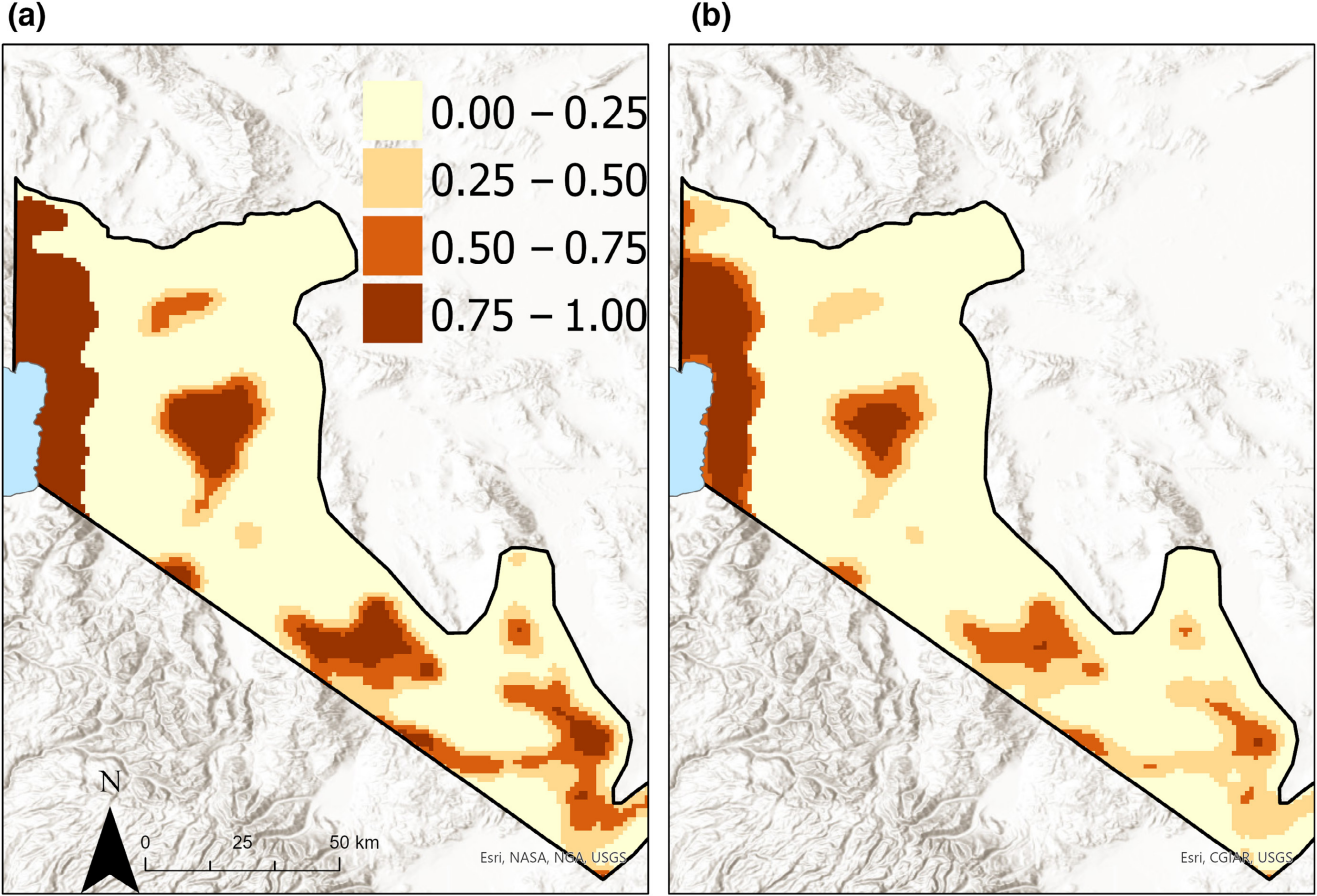
Predicted probability of black bear occupancy (a) and probability of black bear reproduction (cub occupancy) conditional on occupancy by adult bears (b) at a 1‐km^2^ resolution across their established range in the US state of Nevada. At most locations within the Sierra Nevada, both predicted occupancy and reproduction probabilities were high (i.e., >0.50) whereas in the Great Basin, many areas with occupancy probability >0.50 had reproduction probability below 0.50. The maximum predicted probability of reproduction was 0.97. Predictions generated from a multistate occupancy model fit to 3 years of black bear detection data on camera traps, collected from 100 sites, 2018–2020.

Despite low estimated monthly probability of detection for cubs, as a result of the large number of replicates each year at each site (nine), the number of sites estimated in each occupancy state was similar to the proportion we observed occupied (either by cubs or by adults; Table 2). The probability of occupancy for both adult bears and females with cubs was higher in the Sierra Nevada compared to the Great Basin and increased with the proportion of habitat within 5 km of sites (Table 1). When converted to the probability scale, these relationships indicated that adult black bear occupancy probability for sites located in the Sierra Nevada approached 1.0 at sites with proportion habitat (i.e., conifer forest) above 0.25 (Figure 3b). In contrast, estimated adult occupancy probability at sites in the Great Basin was low (<0.40) in landscapes with proportion habitat (i.e., piñon‐juniper woodland) below 0.50 (Figure 3a). Although the estimated relationship between cub occupancy probability and proportion habitat was positive, there was high uncertainty in this relationship, especially in the Great Basin (Figure 3c), despite some cameras in the Great Basin being located in landscapes with the highest proportion habitat (Figure 3c). Using these relationships to predict occupancy and reproduction probabilities across black bear range in Nevada indicated that both adult and cub occupancy probabilities were high in the Sierra Nevada (>0.75, Figure 4a). However, there were multiple areas in the Great Basin with high predicted occupancy of adult black bears (>0.75) but only one continuous area where the predicted probability of cub occupancy (conditional on adult occupancy) was >0.75 (Figure 4b).

**TABLE 2:** Mean estimated number of sites in each of three occupancy states, unoccupied, occupied only by adult bears, and occupied by females with cubs, out of 100 surveyed in each of the three study years.

Occupancy state	2018	2019	2020
Unoccupied	72 (61, 78)	67 (57, 72)	69 (58, 75)
Occupied by adults	14 (6, 24)	18 (11,28)	16 (8, 27)
Occupied by cubs	15 (11, 18)	15 (12, 20)	15 (11, 20)
Total occupied	28 (22, 39)	33 (28, 43)	31 (25, 42)

*Note*: Total occupied refers to the total number of sites estimated occupied by black bears, irrespective of whether cubs were present (i.e., sites occupied by only adults + sites occupied by cubs and adults). 95% credible intervals are shown in parentheses.

## 4 DISCUSSION

Range boundary populations offer opportunities to understand the factors limiting species distributions and forecasting the responses of a species range to climate change (Gaston, 2009). Under the center–periphery hypothesis, declines in habitat quality in peripheral areas of species ranges that reduce population vital rates lead to range boundaries, yet investigations of vital rates at range boundaries for large mammals remain scarce (Pironon et al., 2017). We utilized a strong shift in habitat characteristics, near a regional range boundary to investigate spatial patterns of occupancy and reproduction for American black bears. Our analysis found evidence that habitat at the edge of their range is ecologically marginal and less likely to be occupied by reproductive females compared to core habitat in the region (i.e., the Sierra Nevada), a key prediction of the center–periphery hypothesis. For example, considering sites with 50% habitat within 5 km, estimates of conditional reproduction probability indicate only one third of such sites occupied by adults would also be occupied by cubs in the Great Basin (*R*_*i*_ = 0.32); whereas nearly all such sites in the Sierra Nevada occupied by adults would also be occupied by cubs (*R*_*i*_ = 0.92). Previous analyses focused on spatial patterns of population density in this population likewise demonstrated lower black bear density in piñon‐juniper woodland even after taking into account the amount of habitat (Sultaire et al., 2023). Taken together, these results suggest that habitat on the edge of black bear range in the western Great Basin is ecologically marginal for the species compared to core habitats in the Sierra Nevada. This ecological marginality likely leads to the formation of this range boundary despite the presence of additional habitat, in the form of piñon‐juniper woodland, beyond the range boundary.

There are several possible mechanisms driving lower rates of black bear reproduction in piñon‐juniper woodland compared to mixed‐conifer forest, with differences in primary productivity likely being a strong contributing factor (Gould, Gould, et al., 2019; Welfelt et al., 2019). Reproductive success of carnivorous animals is often closely tied to available resources (Kosterman et al., 2018; Martin et al., 2009). The availability of anthropogenic food subsidies demonstrates reproduction in female black bears is influenced by resource availability (Beckmann & Berger, 2003a), and female black bears without access to high‐quality forage in the fall often fail to produce cubs (Elowe & Dodge, 1989). One of the major food resources likely available to black bears in the Great Basin is piñon nuts, but this resource is temporally variable because single‐leaf piñon is a masting species (Redmond et al., 2012). We do not have estimates of piñon nut production during study years, but such patterns likely influence annual black bear reproduction. Although female bears in eastern forests can compensate for years of scarce resources by switching to alternative food resources (e.g., agricultural subsidies; McDonald Jr & Fuller, 2005), these food resources are largely unavailable in the Great Basin Desert.

Where available, anthropogenic resource subsidies are heavily exploited by black bears in their Nevada range (Beckmann & Berger, 2003b; van Manen et al., 2020), mostly in the more populated urban and exurban areas directly at the interface of the Sierra Nevada and the Great Basin (e.g., Reno, Carson City; Figure 2). Housing densities were slightly elevated within 1 and 5 km of camera locations in the Sierra Nevada compared to the Great Basin, with two outlier sites with respect to housing density in the Sierra Nevada (Figure S2). However, the observed number of adult bears or cubs throughout the study was not similarly elevated at sites with higher housing density, and the number of cub detections was the highest at sites with low housing density in the Sierra Nevada (Figure S3). Additionally, there were a few sites with higher housing density in the Great Basin (Figure S2), but we did not detect cubs in these locations. Thus, observed patterns in black bear reproduction were not likely a result of differences in human resource subsidies, and are more likely related to differences in vegetation characteristics and productivity, between ecoregions.

Elsewhere in American black bear range, lower occupancy of female black bears irrespective of cubs was found at the edge of their range in Minnesota (Ditmer et al., 2018). In that region, bear range abuts extensive croplands that only provides food resources late in the growing season, which male bears can disproportionately access as a result of their higher movement ability compared to females bears (Ditmer et al., 2018). We cannot determine if sex ratios differed near the range boundary from camera data, but this potential similarity between regions with very different ecological characteristics suggests that ecologically marginal conditions (i.e., low habitat quality for females) prevail in the periphery of black bear range. Long‐distance movements by male bears into lower quality habitat at range edges (Ditmer et al., 2018) might explain why support for the center–periphery effects is detectable with black bears whereas many studies of range edges do not find evidence for marginal habitat (Pironon et al., 2017). One limitation of our study was low sample size in core portions of bear range in our study region, with only 11% of study sites located in the Sierra Nevada. More intensive sampling in the Sierra Nevada would have likely reduced uncertainty in our estimates, but the difference in reproductive female occupancy between ecoregions was strong enough to detect despite limited sampling in the Sierra Nevada.

We caution that our multistate occupancy model only quantified the presence of females with cubs, and not necessarily reproductive success. Black bears have a slow life‐history strategy with females needing 2 years to raise cubs to maturity. The presence of especially cubs of the year at sampling sites does not indicate successful reproduction because these cubs may have died before reaching maturity. Although data on cub survival are not available for this population, elsewhere black bear cub mortality in the first year can exceed 40% (Elowe & Dodge, 1989; Garrison et al., 2007) and 60% mortality for the first 2 years (Elowe & Dodge, 1989). Furthermore, across the range of black bears, cub survival was found to be the lowest in the desert southwest (Beston, 2011). However, the highest mortality is often in the first 5 months, and any cubs observed later than July were past this period. Nonetheless, many of the cubs observed on cameras, particularly first‐year cubs, would not ultimately result in successful reproductive attempts. Cub mortality during primary sampling periods could represent a violation of the closure assumption of occupancy models (MacKenzie et al., 2002). However, reproduction does not need to be available for detection during each survey period (Nichols et al., 2007) and our interpretation of reproduction estimates as the probability a site is used at once by black bear cubs during primary sampling periods does not exclude the possibility cubs die before the end of sampling periods. Cub mortality during sampling likely reduced detection probability estimates (for reproduction), as cubs that died were no longer available for detection, which consequently may have contributed to higher uncertainty in estimates of reproduction probability.

Consistent with patterns elsewhere in their range, black bears have expanded their range from the Sierra Nevada into the western Great Basin since the late 1900s (Lackey et al., 2013); however, the geographic extent of their range in Nevada has not changed appreciably since the early 2000s (Lackey, 2004; Sultaire et al., 2023). Lower levels of reproduction in the peripheral areas of their range could contribute to this static range, by lowering the number of potential dispersers that are required to colonize new habitat beyond their current range (Costello et al., 2008). However, immediately beyond their current range lies the Lahontan trough, a very arid portion of the Great Basin with sparse piñon‐juniper woodlands that also inhibits gene flow of other large carnivores (Andreasen et al., 2012). Beyond the trough in central and eastern Nevada lies extensive piñon‐juniper woodlands with evidence of historical occupation by black bears (Lackey et al., 2013). Given this geography, the lower levels of reproduction near the range boundary as well as lower population density previously reported (Sultaire et al., 2023), we conclude that declines in both habitat quality and habitat availability limit the distribution of black bears in the western Great Basin. These declines in habitat quality ultimately prevent black bears from colonizing suitable habitat in central Nevada. This conclusion is consistent with evidence that multiple interacting factors limit the local distribution of mammal species (Sirén & Morelli, 2020).

Our results and previous studies that demonstrated declines in genetic diversity in the Great Basin compared to the Sierra Nevada (Malaney et al., 2018) indicates that this population of black bears generally conforms to the center–periphery hypothesis with ecologically marginal habitats on the range edge. Although the large geographic range of American black bears means that several different factors limit their distribution among regions, throughout much of western North America, black bears inhabit high elevation mountains surrounded by desert (Gould et al., 2022). Piñon‐juniper also occurs in the fringes of these mountains throughout the southwest, suggesting that similar processes, declining reproduction toward the periphery, limit black bear populations elsewhere and not only the availability of habitat. This lower habitat quality in piñon‐juniper woodland should be considered when managing bear populations inhabiting these woodlands and may inhibit the ability of black bears to naturally recolonize further mountain ranges in the Great Basin.

## AUTHOR CONTRIBUTIONS

**Sean M. Sultaire:** Conceptualization (lead); formal analysis (lead); visualization (lead); writing – original draft (lead). **Robert A. Montgomery:** Conceptualization (equal); funding acquisition (equal); investigation (equal); methodology (equal); project administration (equal); resources (equal); supervision (equal); writing – original draft (equal); writing – review and editing (equal). **Patrick J. Jackson:** Conceptualization (equal); funding acquisition (equal); investigation (equal); project administration (equal); resources (equal); writing – original draft (equal); writing – review and editing (equal). **Joshua J. Millspaugh:** Conceptualization (equal); funding acquisition (equal); investigation (equal); methodology (equal); project administration (equal); resources (equal); supervision (equal); writing – original draft (equal); writing – review and editing (equal).

## CONFLICT OF INTEREST STATEMENT

The authors have no competing interests to disclose.

## Supporting information


Appendix S1

## Data Availability

The data that support the findings of this study are openly available in the Dryad Repository at https://doi.org/10.5061/dryad.m905qfv5w.
