# Supplementary material for: Spatial patterns of reproduction suggest marginal habitat limits continued range expansion of black bears at a forest‐desert ecotone
Source: Ecol Evol. 2023 Oct 31;13(11):e10658. doi: 10.1002/ece3.10658 (PMC10616736; doi:10.1002/ece3.10658)
Supplement: Supplementary file 1 — Appendix S1 [file ECE3-13-e10658-s001.docx]

**Supplementary Material**


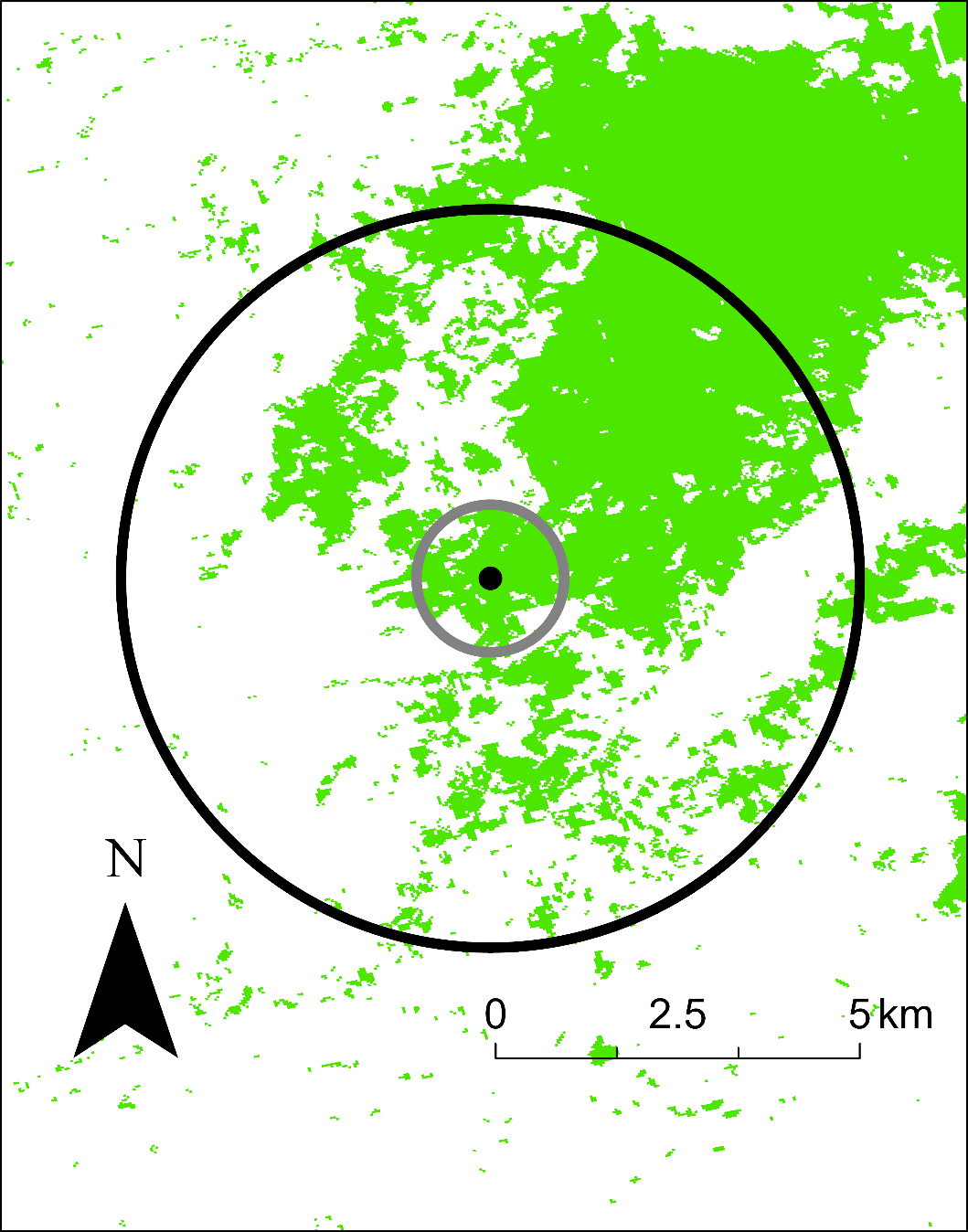


**Figure. S1** Map depicting how we quantified proportion conifer cover (green pixels) surrounding camera trap locations for one randomly selected camera trap location (black point). For occurrence probability of both adult bears and females with cubs, we used a 5-km buffer surrounding sites (black ring) as an occupancy covariate as occupancy represented the probability that a camera location was within a bear’s home range, even on if on the periphery. We used a 1-km buffer (gray ring) as a detection covariate as detection probability represented the intensity of use of a camera location by bears, and hence we predicted it would be more affected by the amount of conifer in the local neighborhood surrounding sites.

**
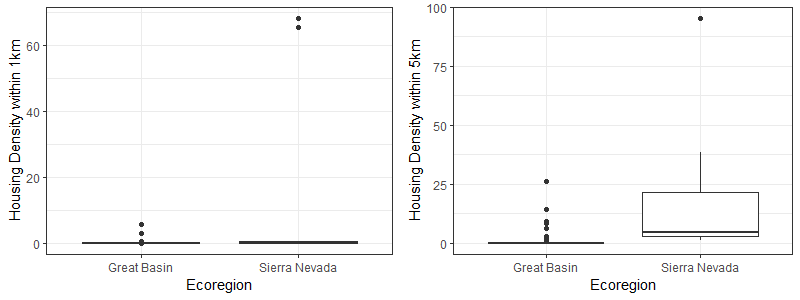
**

**Figure S2**

Box plots showing differences in housing density in landscapes surrounding survey sites between the Sierra Neva and Great Basin ecoregions. The left panel corresponds to housing density within 1km of survey sites, and the right panel plots housing density within 5km of survey sites. Although housing density was on average higher in the Sierra Nevada compared to the Great Basin, particularly at the 5-km scale, these differences did not correlate with where we detected black bears or black bear cubs (see below Figure S3). Housing density was calculated using 2010 data from Census Block Level Housing Change data set available from <https://silvis.forest.wisc.edu/data/housing-block-change/>.

**
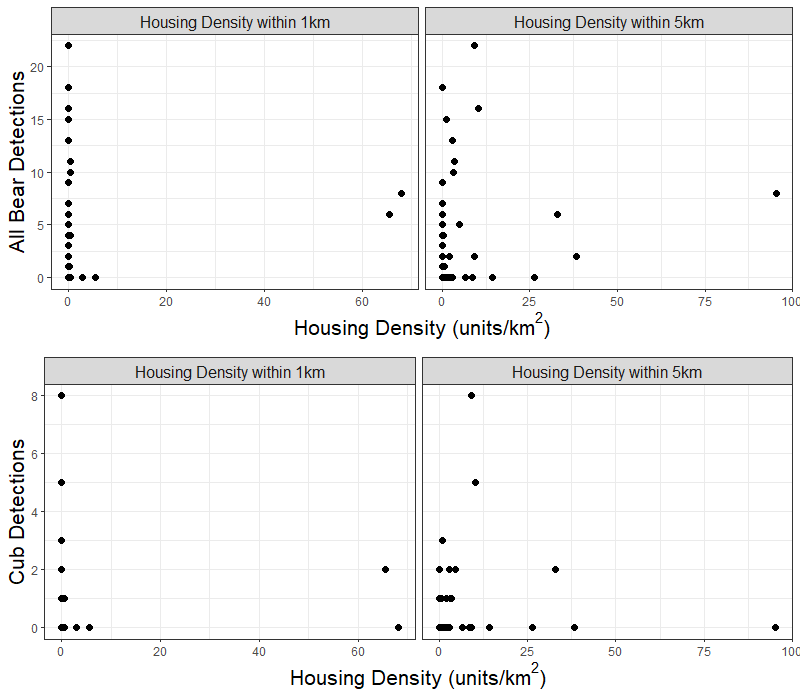
**

**Figure S3**

Relationships between the number of total black bear detections (top panels) and black bear cub detections (bottom panels) and housing density with two different buffer distances of survey sites, 1km (left panels), and 5km (right panels). Housing density was calculated using 2010 data from Census Block Level Housing Change data set available from <https://silvis.forest.wisc.edu/data/housing-block-change/>. Housing density data includes all houses within each census block, including seasonal houses, which could provide anthropogenic to black bears. However, adult black bears and cubs were not detected more often at the few sites with elevated housing density in the surrounding landscape at either spatial scale.

Appendix S1 JAGS code for fitting multi state occupancy model to black bear adult and cubs detections, western Nevada 2018-2020. The code is adapted from Applied Hierarchical Models in Ecology Vol 2: Chapter 6 (Kéry and Royle 2020), accessed from <https://github.com/mikemeredith/AHM_code/blob/main/AHM2_ch06/AHM2_06.4.5.R>.

model{

# Priors and linear models

# (1) State process

# Linear models for annual psi (prob site occupied) and r (prob occupied by cubs, given occupied)

for (i in 1:nsites){

for (t in 1:nyears){

logit(psi[i,t]) <- alpha.lpsi + b.site[site[i]] + beta.lpsi[1] * region[i] +

beta.lpsi[2] * habitat5km[i]

logit(r[i,t]) <- alpha.lr + b.site2[site[i]] + beta.lr[1] * region[i] +

beta.lr[2] * habitat5km[i]

}

}

# Priors for parameters in the linear models of psi and r

### site level random effect on occupancy and reproduction

for (site in 1:nsites){ # Loop over 100 sites

# Intercepts

b.site[site] ~ dnorm(0, tau.site)

b.site2[site] ~ dnorm(0, tau.site2)

}

tau.site ~ dgamma(1.5,.5)

tau.site2 ~ dgamma(1.5,.5)

# Coefficients of 2 covariates

for(k in 1:2){

beta.lpsi[k] ~ dnorm(0, 0.1)

beta.lr[k] ~ dnorm(0, 0.1)

}

###occupancy and reproduction intercepts

alpha.lpsi <- logit(mean.alpha.lpsi)

mean.alpha.lpsi ~ dunif(0, 1)

alpha.lr <- logit(mean.alpha.lr)

mean.alpha.lr ~ dunif(0, 1)

# (2) Observation process

# Linear models in observation process

for (i in 1:nsites){

for(t in 1:nyears){

for(j in 1:nsurveys){

# Observation model for sites in occupied state 1 (= adult bears)

logit(p2[i,j,t]) <- alpha.lp2 + beta.lp2 * conifer1km[i]

# Observation model for sites in occupied state 2 (= cubs)

# Specify linear models

mlogit.p3[2,i,j,t] <- alpha.lp32 + beta.lp32 * conifer1km[i]##assumes det of adults is diff when cubs are present

mlogit.p3[3,i,j,t] <- alpha.lp33 + beta.lp33 * conifer1km[i]

}

}

}

# Priors for parameters in the linear models of p2, p32 and p33

# Intercepts

#for (site in 1:100){

alpha.lp2 <- logit(mean.alpha.p2)

mean.alpha.p2 ~ dunif(0, 1)

alpha.lp32 <- logit(mean.alpha.p32)

mean.alpha.p32 ~ dunif(0, 1)

alpha.lp33 <- logit(mean.alpha.p33)

mean.alpha.p33 ~ dunif(0, 1)

#}

# Coefficients of habitat at 1km on detection

#for(k in 1:2){

#for(reg in 1:2){

beta.lp2 ~ dnorm(0, 0.1)

beta.lp32 ~ dnorm(0, 0.1)

beta.lp33 ~ dnorm(0, 0.1)

#}

#}

# Implement Multinomial logit link for p3[2:3]

for (i in 1:nsites){

for (t in 1:nyears){

for(j in 1:nsurveys){

p3[2,i,j,t] <- exp(mlogit.p3[2,i,j,t]) / (1 + exp(mlogit.p3[2,i,j,t]) +

exp(mlogit.p3[3,i,j,t]))

p3[3,i,j,t] <- exp(mlogit.p3[3,i,j,t]) / (1 + exp(mlogit.p3[2,i,j,t]) +

exp(mlogit.p3[3,i,j,t]))

}

}

}

# Definition of state vector (Omega) and observation matrix (Theta)

# State vector (Omega)

for (i in 1:nsites){

for (t in 1:nyears){

Omega[i,t,1] <- 1 - psi[i,t] # Prob. of unoccupied

Omega[i,t,2] <- psi[i,t] * (1-r[i,t]) # Prob. of occ. by adult bear

Omega[i,t,3] <- psi[i,t] * r[i,t] # Prob. of occ. by cubs

}

}

# Observation matrix (Theta)

# Order of indices: true state, observed state, site, occasion, year

for(i in 1:nsites){

for (t in 1:nyears){

for (j in 1:nsurveys){

Theta[1,1,i,j,t] <- 1

Theta[1,2,i,j,t] <- 0

Theta[1,3,i,j,t] <- 0

Theta[2,1,i,j,t] <- 1-p2[i,j,t]

Theta[2,2,i,j,t] <- p2[i,j,t]

Theta[2,3,i,j,t] <- 0

Theta[3,1,i,j,t] <- 1-p3[2,i,j,t]-p3[3,i,j,t]

Theta[3,2,i,j,t] <- p3[2,i,j,t]

Theta[3,3,i,j,t] <- p3[3,i,j,t]

}

}

}

# State-space likelihood

# State equation: model of true states (z)

for (i in 1:nsites){

for (t in 1:nyears){

z[i,t] ~ dcat(Omega[i,t,])

}

}

# Observation equation: model for observed multistate detections

for (i in 1:nsites){

for (t in 1:nyears){

for (j in 1:nsurveys){

y[i,j,t] ~ dcat(Theta[z[i, t], ,i,j,t])

}

}

}

# Derived quantities

# Number of sites in each state per year

for (t in 1:nyears){

for (i in 1:nsites){

state1[i,t] <- equals(z[i,t], 1) # Indicator for site in state 1

state2[i,t] <- equals(z[i,t], 2) # ... state 2

state3[i,t] <- equals(z[i,t], 3) # ... state 3

stateOcc[i,t] <- max(state2[i,t], state3[i,t]) # ... occupied site

}

n.occ[t,1] <- sum(state1[,t]) # Number of unoccupied sites

n.occ[t,2] <- sum(state2[,t]) # Number of sites with adult bears

n.occ[t,3] <- sum(state3[,t]) # Number of sites with cubs

n.occ.total[t] <- sum(stateOcc[,t]) # All occupied

}

}
